# Venous Malformations and Blood Coagulation in Children

**DOI:** 10.3390/children8040312

**Published:** 2021-04-20

**Authors:** Johanna Aronniemi, Satu Långström, Katariina A. Mattila, Anne Mäkipernaa, Päivi Salminen, Anne Pitkäranta, Johanna Pekkola, Riitta Lassila

**Affiliations:** 1 University of Helsinki, Yliopistonkatu 4, 00100 Helsinki, Finland; satu.langstrom@hus.fi (S.L.); katariina.mattila@helsinki.fi (K.A.M.); anne.makipernaa@hus.fi (A.M.); paivi.salminen@hus.fi (P.S.); anne.pitkaranta@hus.fi (A.P.); johanna.pekkola@hus.fi (J.P.); riitta.lassila@hus.fi (R.L.); 2 HUS Diagnostic Center, Department of Radiology, New Children’s Hospital, Helsinki University Hospital, P.O. Box 347, Stenbäckinkatu 9, 00029 Helsinki, Finland; 3 VASCERN VASCA European Reference Centre, P.O. Box 347, Stenbäckinkatu 9, 00029 Helsinki, Finland; 4 Division of Hematology-Oncology and Stem Cell Transplantation, New Children’s Hospital, Helsinki University Hospital, P.O. Box 347, Stenbäckinkatu 9, 00029 Helsinki, Finland; 5 Department of Pediatric Surgery, New Children’s Hospital, Helsinki University Hospital, P.O. Box 347, Stenbäckinkatu 9, 00029 Helsinki, Finland; 6 Coagulation Disorders Unit, Department of Hematology, Comprehensive Cancer Center, Helsinki University Hospital, P.O. Box 372 Haartmaninkatu 4, 00029 Helsinki, Finland; 7 Department of Otorhinolaryngology-Head and Neck Surgery, Helsinki University Hospital, Kasarmikatu 11-13, P.O. Box 263, 00029 Helsinki, Finland; 8 HUS Diagnostic Center, Department of Radiology, Töölö Hospital, P.O. Box 266, 00029 Helsinki, Finland

**Keywords:** children, coagulation disturbances, coagulation factors, D-dimer, venous malformation

## Abstract

Introduction: Venous malformations (VMs) are congenital low-flow lesions with a wide spectrum of clinical manifestations. An increasing number of studies link VMs to coagulation abnormalities, especially to elevated D-dimer and decreased fibrinogen. This condition, termed localized intravascular coagulopathy (LIC), may pose a risk for hemostatic complications. However, detailed data on the laboratory variables for coagulation and fibrinolytic activity in VM patients are limited. We addressed this question by systematically analyzing the coagulation parameters in pediatric VM patients. Methods: We included 62 patients (median age 11.9 years) with detailed laboratory tests for coagulation and fibrinolytic activity at a clinically steady phase. We assessed clinical and imaging features of VMs and their correlations with coagulation and fibrinolysis variables using patient records and MRI. Results: D-dimer was elevated in 39% and FXIII decreased in 20% of the patients, as a sign of LIC. Elevated D-dimer and decreased FXIII were associated with large size, deep location, and diffuse and multifocal VMs. FVIII was elevated in 17% of the patients and was associated with small VM size, superficial and confined location, discrete morphology, and less pain. Surprisingly, antithrombin was elevated in 55% of the patients but without associations with clinical or other laboratory variables. Conclusions: LIC was common in pediatric patients with VMs. Our results provide a basis for when evaluating the risks of hemostatic complications in children with VMs. Further research is warranted to explore the mechanisms behind coagulation disturbances and their relation to clinical complications.

## 1. Introduction

Venous malformations (VMs) are congenital developmental defects of the post-capillary vasculature with low-flow characteristics [1]. They can be local, multifocal, or diffuse and may invade superficial, deep, or visceral tissues in any part of the body.

Patients with VMs commonly have abnormalities in blood coagulation. If overlooked and left untreated, coagulopathy may increase morbidity and treatment complications. Thus, the diagnostic workup of VMs includes a baseline evaluation of blood cell count (BCC) and coagulation status to identify patients in need of management and follow-up of coagulopathy.

The association between VMs and coagulopathy is established; D-dimer has been reported to be elevated in 40–60% of VM patients in various study populations [2,3,4,5]. This condition, termed localized intravascular coagulopathy (LIC) [4], manifests as systematically elevated fibrin degradation products in the circulation. In severe LIC, fibrinogen and platelet counts are concomitantly decreased (4). LIC is associated with recurrent local thrombosis and formation of small intravascular calcifications (phleboliths). Following trauma, infection, surgery, or sclerotherapy, LIC may progress to systemic disseminated intravascular coagulopathy (DIC), in which in addition to changes in severe LIC, prothrombin time is prolonged. In DIC, the regulation of coagulation has failed predisposing to life-threatening local or systemic coagulation abnormalities [6,7,8]. Patients with LIC have been found to get benefit from subcutaneous low-molecular-weight heparin (LMWH) [5,7,9] together with any management tools decreasing the volume of the malformation, such as compression garments, sclerotherapy, or surgical resection.

Despite the growing awareness of coagulation disorders in VM patients and knowledge of interactions between angiogenesis and coagulation [10], data on the associations between VMs and coagulation biomarkers beyond D-dimer and fibrinogen are scarce. In our hospital, we have routinely screened coagulation and fibrinolysis activity as well as blood cell count in pediatric VM patients for over a decade. In this study, we retrospectively analyzed these results and report the associations between malformation-related variables and coagulation biomarkers.

## 2. Materials and Methods

This retrospective study was carried out at Helsinki University Hospital (HUH), New Children’s Hospital, in the Divisions of Pediatric Surgery, Pediatric Radiology, and Hematology. HUH has had an interdisciplinary team for vascular anomalies since 2002 to serve as a tertiary referral center for vascular anomalies for the whole country. HUH’s institutional review board approved the study protocol.

We conducted an automated search for vascular anomaly patients (based on ICD codes) from the outpatient visit registry of our pediatric surgery clinic for a 14-year period (from 2002 to 2015), resulting in a cohort of 434 patients with miscellaneous vascular malformations. We analyzed all MRI studies and patient records to identify patients with VMs (*n* = 133). In addition to common VMs, we included combined vascular malformations with a substantial venous component, such as capillary–venous malformations (CVMs) and capillary–lymphatic–venous malformations (CLVMs). We excluded patients whose laboratory results, obtained on a clinical basis, did not cover both the detailed analysis of coagulation and fibrinolysis activity and blood cell count. All the laboratory analyzes were conducted at the same time point of a steady clinical stage and before initiation of any invasive procedures or anticoagulation therapy. The final study population comprised 62 pediatric patients. We obtained information on age, gender, pretreatment symptoms, subsequent anticoagulation therapy, and complications from the patient records. The symptoms (pain, swelling, functional impairment) were mostly registered by the same physician (P.S.) and recorded as presence or absence of each of them.

**Figure 1:**
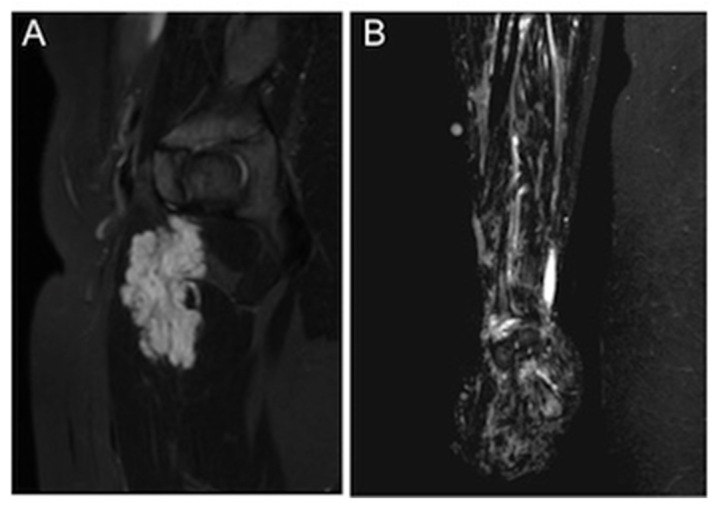
An example of the two different morphological appearances of VMs in MRI. (**A**) A discrete and well-demarcated VM in the thigh muscles. (**B**) A diffuse VM of the right forearm.

We determined the size, location, affected tissue planes, morphological appearance, and presence of phleboliths of each VM based on the MRI studies. Because VMs are irregularly shaped and often diffusely infiltrate normal tissues, exact dimensions could not be obtained. We measured the greatest diameter in any of the three planes and graded it as small (<5 cm), medium (5–10 cm), wide (10–25 cm), or extensive (>25 cm). Lesion location was defined as head/neck, trunk, or extremities. According to the affected tissue planes, the depth of the lesion was categorized into three groups: (1) superficial lesions confined to subcutaneous fat, (2) lesions with an intramuscular, -osseal, or -articular component, and (3) deep lesions with an intra-abdominal, retroperitoneal, or intra-thoracic component. We divided the malformations into two categories by morphology: (A) discrete, relatively well-demarcated malformations and (B) diffuse, phlebectatic malformations (Figure 1). Phleboliths were defined as small intraluminal foci of signal-voids, visible both in T1- and T2-weighted sequences, consistent with calcifications.

Laboratory analyses included plasma prothrombin time (PT, %, Nycotest PT, Axis-Shield PoC As), activated partial thromboplastin time (APTT, s, Actin FSL, Dade Behring), thrombin time (TT, s, BC Thrombin Reagent, Siemens Healthcare Diagnostics), antithrombin (AT, %, chromogenic assay, Berichrom. Antithrombin III, Dade Behring), fibrinogen (modified Clauss method, Multifibren U, Siemens Healthcare Diagnostics), coagulation factor VIII (FVIII) activity (FVIII:C, one-stage clotting assay, Pathromtin SL, Dade Behring and FVIII Deficient Plasma), von Willebrand factor antigen (VWF:Ag with VWFag Latex Reagent, Grifols), and ristocetin cofactor activity (VWF:RCo with BC von Willebrand Reagent, all analyzed with the BCS XP analyzer, Siemens Healthcare Diagnostics), FV, VII, IX, and X activities (measured by prothrombin time-based clotting assays utilizing commercial factor-deficient plasmas), FXIII (FXIII) activity (Berichrom FXIII, Dade Behring, Siemens), D-dimer (immunoturbidimetric assay, Tina-quant D-dimer, Roche Diagnostics), lupus anticoagulant (LA, two screening tests based on APTT; IL Test APTT-SP, Instrumentation Laboratory and Russell Viper Venom activated clotting time, DVVtest 10, (American Diagnostica, Inc., Stamford, CT United States)

For the blood cell count, we applied the common age-adjusted reference limits of our laboratory. For coagulation biomarkers and coagulation times, we used adult reference limits, because adjusted limits for pediatric patients were not available in our laboratory. The reference limits were as follows: PT 70–130%, APTT 23–40 s, TT 17–25 s, antithrombin 84–108%, fibrinogen 1.7–4.0 g/L, FV 79–128 IU/dL, FVII 76–170 IU/dL, FVIII 52–148 IU/dL, FIX 67–135 IU/dL, FXIII 76–156 IU/dL, protein C 74–141%, protein S 66–150%, VWF:Ag 50–169%, VWF:RCo 44–183%, and D -dimer < 0.5 mg/L.

### Statistics

An independent statistician performed the statistical analyses (NCSS 8, NCSS, LLC, Kaysville, UT, USA, www.ncss.com (accessed on 1 November 2017). For the calculation of correlations between coagulation markers, power transformations were used for non-parametric variables. Parametric and transformed non-parametric variables were compared with Pearson’s correlation analysis (r) and non-parametric variables with Spearman’s rank correlation coefficient (ρ). All correlations were verified with scatter plots.

Chi-square test or Fisher’s exact test served for paired comparisons between clinical and MRI findings (morphology, diagnosis, location, depth, phleboliths, age, gender) and coagulation markers that were most commonly (>10% of patients) deviating from the reference limits (FV < 79 IU/dL, FVII < 76 IU/dL, FVIII > 148 IU/dL, FXIII < 76 IU/dL, AT3 > 108%, thrombin time < 17 s, and D-dimer > 0.5 mg/L). Mann–Whitney U-test was used for paired comparisons of non-parametric variables. We considered *p*-values < 0.05 as statistically significant.

## 3. Results

### 3.1. Patient Characteristics

Our study consists of 62 pediatric patients with a median age of 11.9 years at the time of laboratory analysis and a female-to-male ratio of 1.3. MRI examination (1.5T) with gadolinium enhancement was performed in all malformations. The size of the malformations varied from small to extensive (>25 cm) lesions. Most of the VMs located in the extremities (71%) (Table 1). Phleboliths, visible in MRI, were present in 29 malformations (47%). Majority of the patients had a common VM (89%), whereas seven patients had a combined vascular malformation with a significant venous component (Table 1). The most common symptom at the time of initial evaluation was pain, affecting 81% of patients. The proportions of swelling and functional impairment were 67 and 25%, respectively.

### 3.2. Blood Cell Count

Hemoglobin was within the reference range in all but one patient. Significant thrombocytopenia was absent. Leukocytes were slightly decreased in 33% of patients (<5.0 × 10^9^/L, median 5.8 × 10^9^/L).

### 3.3. Coagulation Screening Tests and D-Dimer

**Figure 2:**
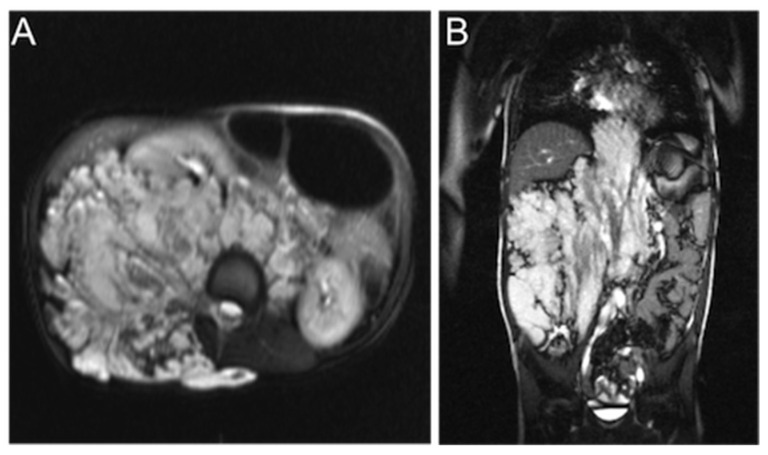
Axial (**A**) and coronal (**B**) T2-weighted MR images of an extensive VM affecting the retroperitoneum and spinal muscles and dislocating the bowel and the right kidney to the left.

**Figure 3:**
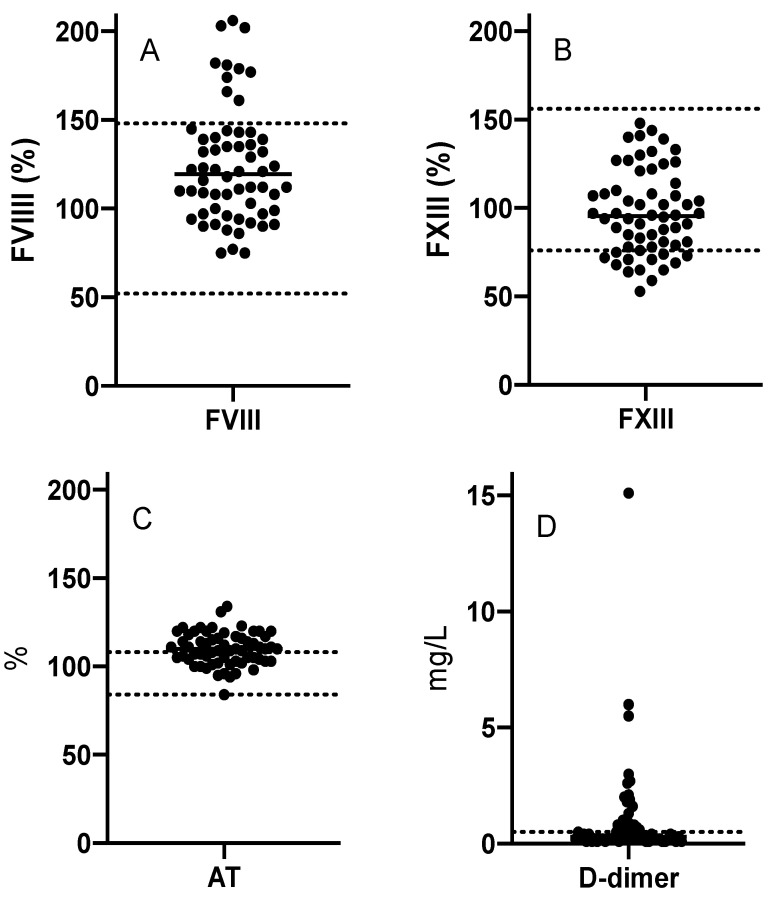
Coagulation factors VIII (**A**) and XIII (**B**), antithrombin (**C**), and D-dimer (**D**) in patients with venous malformatons. Dotted lines depict reference ranges for each variable.

Prothrombin times ranged from 64 to 157%, thus being normal for the age of the patients. APTT was prolonged (>40 s) in two patients. Shortened APTTs, compatible with contact activation, did not emerge in our VM patients. Thrombin time was short (<17 s) in six (10%) but prolonged (>25 s) in one patient with an extensive retroperitoneal and intramuscular VM (Figure 2). This patient had concomitantly prolonged APTT, high D-dimer, and very low fibrinogen compatible with prolonged thrombin time. All in all, D-dimer was elevated (≥0.5 mg/L) in 39% of patients, indicating enhanced systemic fibrin turnover (mean 1.0 mg/L, range 0.1–15.1 mg/L) (Figure 3, Table 2).

### 3.4. Coagulation Factors and Antithrombin

In most patients, coagulation factors were within adult reference ranges. However, FXIII activity was decreased in 20% and FV activity in 10% of the patients (Figure 3, Table 2). FVIII activity was elevated in 17% of the patients (Figure 3, Table 2). Fibrinogen was undetectably low in the above-mentioned patient with the highest D-dimer (15 mg/L) (Figure 2). The other patients had normal or elevated fibrinogen levels. Coagulation factors did not significantly associate with each other or with D-dimer. Vitamin K-dependent FVII level correlated with PT (ρ = 0.71, *p* < 0.0001) and protein C (ρ = 0.50, *p* < 0.0001), as anticipated. Although FVIII was commonly elevated (17%), its carrier protein, VWF, was elevated in only two patients (3.8%). The proportion of patients with elevated antithrombin was notable (55%) (Figure 3, Table 2), but it did not correlate with the clinical features or with other hemostatic factors.

### 3.5. Associations with the Clinical Features of Malformations

**Figure 4:**
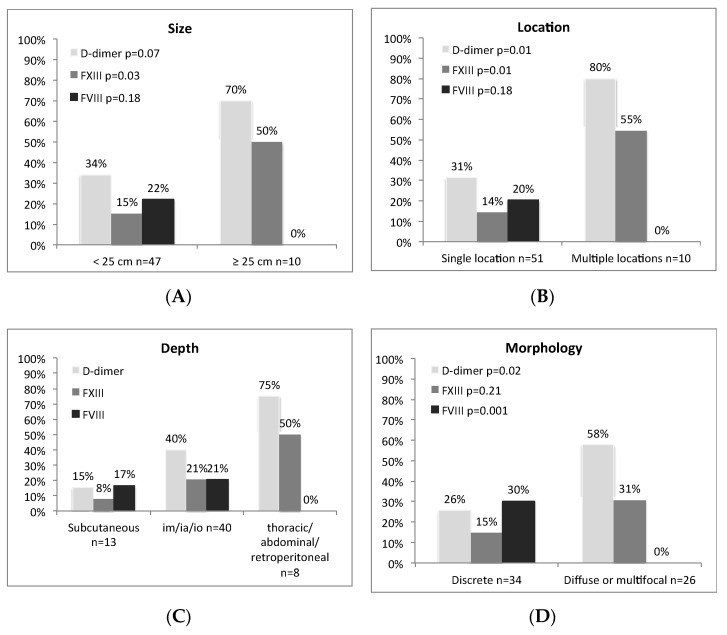
Associations between coagulation variables and clinical and imaging findings. The columns indicate the percentage of patients whose coagulation variables were outside the reference limits (elevated FVIII > 148 IU/dL, decreased FXIII < 76 IU/dL, and elevated D-dimer ≥ 0.5 mg/L). (**A**) Lesion size; comparison of the largest size group (>25 cm) with the smaller size group. (**B**) Lesion location; single location lesions are limited to one location (head/neck, extremities, or trunk), multiple locations extend to more than one location or indicate multiple lesions in different locations. (**C**) Lesion depth; a comparison between 1. subcutaneous, 2. muscle-, joint-, and/or bone-affecting, and 3. intrathoracic, intra-abdominal, or retroperitoneal lesions. The *p*-values are non-applicable in **C** due to too small groups for the Chi-square test. (**D**) MRI-based morphology; comparison between discrete, well-demarcated lesions and diffuse or multifocal lesions.

D-dimer, FVIII, and FXIII were significantly associated with the clinical features of the malformations. Elevation of D-dimer and decrease in FXIII were most common in combined, deep, wide, and diffuse/multifocal lesions as well as in lesions extending to multiple locations (Figure 4). D-dimer was rarely elevated in the smallest (<5 cm) lesion group (*p* = 0.02) and in lesions in the most superficial locations (*p* < 0.05).

FVIII, instead, was not elevated in diffuse/multifocal or deeply seated malformations, or in malformations covering multiple locations or with a diameter greater than 25 cm (Figure 4). Elevated FVIII was associated with less reported pain (*p* = 0.02), independent of the presence of phleboliths, and with female gender (*p* = 0.01). Other coagulation markers, including D-dimer, did not correlate with any of the symptoms.

No significant correlations emerged between the coagulation biomarkers and the presence of phleboliths in MRI, patient age, gender, or the location (extremities, trunk, or head/neck) of the malformations.

### 3.6. Complications

Complications related to hemostatic disorders during the study period (2002–2015) were few; two patients suffered from a spontaneous local bleeding episode, and one of them also had a severe bleeding complication during sclerotherapy for retroperitoneal VM. Additionally, one patient with intra-articular VM of the knee developed cartilage damage, presumably due to intra-articular bleeds. All patients suffering from bleeding complications had elevated D-dimer. Neither thrombotic nor bleeding events occurred outside the malformations.

### 3.7. Anticoagulant Therapy

Patient were not on anticoagulant therapy at the time of coagulation assessments. Eight patients (13%) later received LMWH and two patients were managed with acetylsalicylic acid. Three patients were on continuous LMWH, while for five, the therapy was periodic. The indications for LMWH therapy were surgery or sclerotherapy under elevated D-dimer (6), constantly elevated D-dimer (3), pain (3), thrombophlebitis (3), and severe coagulopathy (1), some patients having several indications. All patients who received LMWH for pain reported a reduction in pain after the therapy was commenced, and the coagulation variables improved for the patient with severe coagulopathy (data not shown).

## 4. Discussion

We aimed to analyze coagulation variables in pediatric patients with VMs to better evaluate risks for hemostatic complications in this patient group. These congenital lesions, with low-flow characteristics and highly variable clinical manifestations, are known to be associated with coagulation abnormalities, but the previous analyses on coagulation status have mainly been carried out in adult patients [4,5]. The coagulation system in children is known to differ from adults, and thus, the knowledge of the possible coagulation abnormalities in this particular group is of importance.

D-dimer was elevated in over one-third (39%) of these pediatric patients with VMs. Additionally, coagulation factors FVIII and FXIII were abnormal in 20% of the patients, with FXIII being decreased but FVIII elevated. Specifically, D-dimer, FVIII, and FXIII correlated significantly with clinical and morphological features of the malformations. Antithrombin was elevated in over half (55%) of the patients but without clinical correlations.

The pathogenesis of coagulopathy in VM patients is not fully understood. According to the theory of LIC, coagulation and subsequent fibrinolysis constantly occur in the dilated venous spaces, leading to consumption of coagulation factors [4,5,7]. However, the concept of LIC is only applied in the context of VMs [2,3,4,5,11]. It relies on findings in peripheral blood, not on local blood sampling and nevertheless deems the coagulopathy as local. However, this unique local disturbance in coagulation status may affect regulation of the coagulation system and, thus, affect the systemic risk of bleeding and thrombosis.

Detailed reports on coagulation system disturbances in VM patients are scarce, despite growing evidence linking elevated D-dimer to VMs [2,3,4,5,11]. In previous studies in adults, 40–60% of the patients showed elevated D-dimer [4,5], and in a study of 24 pediatric patients predominantly with head and neck VMs, 33% had elevated D-dimer [3]. In the current larger study of pediatric patients, the proportion of patients with elevated D-dimer is similar to other studies in both children and adults. These results indicate enhanced fibrin turnover in these congenital lesions, not only upon disease progression at adulthood, but already at an early age.

We found no previous reports on coagulation factors, except fibrinogen and von Willebrand factor, in VM patients, although reduced activities of FV, FVIII, FXIII, and antithrombin in addition to fibrinogen and vWf in severe LIC have been speculated [7]. Fibrinogen was decreased in only one of our patients, whereas previous studies in varying VM populations report low fibrinogen in up to 10% of patients [4,5].

FVIII was elevated in 17% of the VM patients in our study. An elevated level of FVIII has been linked to venous and arterial thrombosis as well as to recurrence of venous thrombosis both in adults and children [12,13]. In this study, however, no thrombosis outside the malformations occurred. The carrier protein of FVIII, VWF, was within the normal range in almost all patients; binding of FVIII to platelet-derived microparticles is one possible explanation for this discrepancy [14]. Previously, VWF activity was low in 27% and notably low (<50%) in 12% of 118 trunk and extremity VM patients, being mainly adults [5]. VWF is linked to angiogenesis, and low activity of VWF has been associated to the development of vascular malformations [15]. This association could not be confirmed in this study of pediatric patients only.

The activity of FXIII is essential for clot stability. When FXIII activity declines, as in many of our VM patients, the forming fibrin network becomes more loose and susceptible to fibrinolysis [16,17,18]. In addition, a recent study shows that the VM-causative TIE2 mutations cause significant changes to the endothelial morphology and activation of the plasminogen/plasmin protease system. This mechanism further enhances fibrinolysis and increases the D-dimer level [19,20]. On the other hand, impaired fibrin polymerization enhances thrombin generation when FXIII activity is low. These findings warrant more attention in future studies, also on the role of FXIII.

Antithrombin, besides inactivating coagulation factors, carries anti-inflammatory and antiangiogenic effects [21]. During DIC and sepsis, antithrombin activity typically decreases due to increased consumption [22]. Low antithrombin activity undisputedly predisposes to venous thrombosis, but little is known about its increased activity [22,23]. We assume that elevated antithrombin protects from excessive coagulation activity in VMs, but its true impact remains unclear. Additionally, whether this phenomenon of increased antithrombin activity is unique to pediatric patients only is not known.

Coagulation variables also correlated with clinical features of the malformations. D-dimer was elevated in up to 75% of those patients with deep malformations and in up to 80% of the patients with multiple malformations. Additionally, low FXIII was observed in around 50% of these patients, both findings implying that the patients with deep or multiple malformations may be more susceptible to hemostatic complications. In the current patient group, hemostatic complications were, however, uncommon.

Limitations of the study included retrospective study design, relatively small patient numbers regarding the evaluation of clinical symptoms, associations between coagulation variables, and different VM subgroups. We utilized adult reference values for the coagulation biomarkers because age-adjusted values for children were not locally available. However, the variables that were most commonly outside reference ranges (D-dimer, FV, FVIII, FXIII, antithrombin) are at adult levels from birth or from early childhood [21,24,25], and thus, adult reference ranges could be applied.

## 5. Conclusions

Our findings reveal associations between VMs and the coagulation variables, which are also key players in angiogenesis. Whether they are manifestations of genetically regulated physiological status leading to vascular pathology, or vice versa, is unknown. Further studies on expression and co-regulation of angiogenesis and coagulation are needed to better understand the pathophysiology of VM-related coagulopathy and to facilitate its treatment [26].

## Figures and Tables

**Table 1:** Patient characteristics.

	N (%) or Median (Range)
**Patients**	62
Male	27 (44)
Female	35 (56)
Age	11.4 (1.1–19.4)
**Lesion size (cm) (excluding multifocal lesions)**	
<5	15 (24.2)
5–10	18 (29)
10–25	14 (22.6)
>25	10 (16.1)
**Lesion morphology**	
Multifocal	5 (8.1)
Diffuse, infiltrating	22 (35.5)
Discrete, well-demarcated	35 (56.5)
**Lesion location**	
Head and neck	2 (3.2)
Trunk	5 (8.1)
Extremities	44 (71.0)
Multiple locations	11 (17.7)
**Lesion depth**	
Subcutaneous only	13 (21.0)
ia/io/im component	40 (64.5)
abd./retroperit./thoracic	9 (14.5)
**Diagnosis**	
VM	55 (88.7)
Combined:	7 (11.3)
LVM	4 (6.5)
CVM	2 (3.2)
LCVM	1 (1.6)

CVM = capillary-venous malformation, ia = intra-articular, im = intramuscular, io = intraosseal, LCVM = lymphatic-capillary-venous malformation, LVM = lymphatic-venous malformation, VM = venous malformation.

**Table 2:** Abnormalities in blood cell count, coagulation factors, and natural anticoagulants.

Biomarker	Reference Limit	N	Median	Range	Values below Reference Range N (%)	Values above Reference Range N (%)
Leukocytes (E9/L)	5–14	57	5.8	2.8–14.9	19 (33.3)	1 (1.8)
FV (IU/dL)	79–128	61	102	56–136	6 (9.8)	2 (3.3)
FVIII (IU/dL)	52–148	60	119.5	75–206	0	10 (16.7)
FXIII (IU/dL)	76–156	60	95.5	53–141	12 (20.0)	0
Antithrombin (%)	84–108	62	110.0	84–134	0	34 (54.8)
D-dimer (mg/L)	<0.5	61	0.3	0.1–15.1	0	24 (39.3)
Fibrinogen (g/L)	1.7–4	60	2.85	0.0–5.2	1 (1.7)	6 (10.0)
VWF:RCo (%)	44–183	53	82.0	44–220	0	2 (3.8)

## Data Availability

The data presented in this study are available on request from the corresponding author. The data are not publicly available due to protection of patient privacy.

## Abbreviations

BCC = blood cell count, CVM = capillary–venous malformation, CLVM = capillary–lymphatic–venous malformation, DIC = disseminated intravascular coagulation, F = coagulation factor, LIC = localized intravascular coagulopathy, LMWH = low-molecular-weight heparin, LVM = lymphatic-venous malformation, VM = venous malformation.
